# World’s Fair at New York

**Published:** 1853-01

**Authors:** 


### WORLD'S FAIR AT NEW YORK.

We believe it is the wish of many in the profession, that Dentistry—at least so far as Instruments are concerned—shall be well represented at this exhibition.

It has always appeared to us, that in one thing our profession is somewhat deficient, we mean standard form or pattern for certain instruments—those most generally used by the profession. Our cutlers complain of this, and say, that no two want the same kind of instrument. Now this is not so much the case in general Surgery. A well adapted forcep should suit nine out of ten practitioners. A few of the most desirable plugging instruments should suit almost every dentist. But such is not the case. We have never seen even among the largest assortment of instruments all the points most desirable for filling teeth and compressing the gold &c. For instance, could any of our old operators go into the most complete dental establishment in the United States or the World, and select a complete set of instruments with which he would operate? would he not have to change at least half the points? We do not regard this as any fault of the cutlers, and it may be owing in part to the progressive state of the profession; yet we think it time to alter this condition of things. We feel assured that the profession as well as our instrument makers, would be benefited by this change, having so far as is possible, a standard form for all of the instruments most generally used. Long habit, we know, does often accustom us to defects in an instrument, which would at first appear insurmountable; and hence, rather than accustom ourselves to a far better instrument, we may hold on to the *familiar one*; but this is not right, for with the better one we may be able to do better justice to our patient. We go in for improvement, and hope we shall ever be willing to try that which holds out this prospect.

In relation to the *fair*, one of our correspondents holds the following language; "I am a little solicitous that the dental department at the *world's* fair in New York, next May, shall be handsomely, or to say the least of it, usefully represented in every particular, not so much in teeth or plate work, as in that which will benefit most of us, I mean the operative and surgical, the most simple, compact, complete and useful instruments, as to form, size, shape &c., from an Elevator (as a Caldwell) up to a Holder-Still of a woman's tongue while filling a lower molar. A complete set of dental instruments might be collected together at that fair, that would be esteemed more useful and *universally adopted* by the *profession*. I make the suggestion for your movement or consideration; for instance, let every dentist of experience send to that fair some *one* or more instruments (if he has any) that he made, or order-made under his direction, that he considers better than any other he ever saw for the same purpose, by which means we can select the most complete instruments in each and every department of the profession; and as a double incentive let the profession offer an extra premium, and the funds be raised by dentists who are enjoying a fair practice, each contributiug say, Five or more dollars to some competent committee, raised for the purpose of investigating, awarding, etc.."

The above plan if carried out, would we think enlist a great deal of competition, and be productive of good. We would have no objections to see the different styles of mechanical work presented, although we have but a poor opinion of dental specimens prepared *in fancy* style for the mouth, and which may attract much attention from the uninitiated, yet which never would do service in the mouth. We have had enough of this kind of exhibition; and we never could consent to enter the list after this fashion. But our correspondent goes in for the useful, and so far we second the motion, and hope if the proposition meets favor, it will be responded to by the profession, east and west.

